# Explainable self-supervised learning for medical image diagnosis based on DINO V2 model and semantic search

**DOI:** 10.1038/s41598-025-15604-6

**Published:** 2025-09-01

**Authors:** Alaa Hussien, Abdelkareem Elkhateb, Mai Saeed, Nourhan M. Elsabawy, Alaa Ebraheem Elnakeeb, Nora Elrashidy

**Affiliations:** 1 Machine Learning and Information Retrieval Department, Faculty of Artificial Intelligence, Kaferelshikh University, Kaferelshikh, 33511 Egypt; 2 Biological Artificial Intelligence Program, Faculty of Artificial Intelligence, Kafer Elsheikh University, Kaferelshikh, Egypt

**Keywords:** DINO, Explainability, Self-supervised learning, Semantic search, Medical imaging, Health care, Nanoscience and technology

## Abstract

Medical images have become indispensable for decision-making and significantly affect treatment planning. However, increasing medical imaging has widened the gap between medical images and available radiologists, leading to delays and diagnosis errors. Recent studies highlight the potential of deep learning (DL) in medical image diagnosis. However, their reliance on labelled data limits their applicability in various clinical settings. As a result, recent studies explore the role of self-supervised learning to overcome these challenges. Our study aims to address these challenges by examining the performance of self-supervised learning (SSL) in diverse medical image datasets and comparing it with traditional pre-trained supervised learning models. Unlike prior SSL methods that focus solely on classification, our framework leverages DINOv2’s embeddings to enable semantic search in medical databases (via Qdrant), allowing clinicians to retrieve similar cases efficiently. This addresses a critical gap in clinical workflows where rapid case The results affirmed SSL’s ability, especially DINO v2, to overcome the challenge associated with labelling data and provide an accurate diagnosis superior to traditional SL. DINO V2 provides 100%, 99%, 99%, 100 and 95% for classification accuracy of Lung cancer, brain tumour, leukaemia and Eye Retina Disease datasets, respectively. While existing SSL models (e.g., BYOL, SimCLR) lack interpretability, we uniquely combine DINOv2 with **ViT-CX**, a causal explanation method tailored for transformers. This provides *clinically actionable* heatmaps, revealing how the model localizes tumors/cellular patternsa feature absent in prior SSL medical imaging studies Furthermore, our research explores the impact of semantic search in the medical images domain and how it can revolutionize the querying process and provide semantic results alongside SSL and the Qudra Net dataset utilized to save the embedding of the developed model after the training process. Cosine similarity measures the distance between the image query and stored information in the embedding using cosine similarity. Our study aims to enhance the efficiency and accuracy of medical image analysis, ultimately improving the decision-making process.

## Introduction

In the healthcare domain, Medical imaging technologies have become essential, playing a vital role in decision-making and treatment planning. The steady increase in medical imaging in modern healthcare shows the significance of medical imaging. However, with this expansion comes a new challenge: the gap between the increasing number of medical images and the available radiologists has increased. This gap has led to a noteworthy rise in the workload of radiologists, forcing them to work extremely fast. This immense workload has resulted in delays and human errors in diagnosis, creating an urgent need to include automated systems to improve diagnostic accuracy^1,2^. Recent research emphasizes the potential of deep learning models in medical image diagnosis, illustrating expert-level accuracy. However, these models encounter noteworthy challenges, mainly due to their dependence on large labelled datasets, which are costly and time-consuming to create because of the specialized expertise required for annotation. Furthermore, the interpretability of these models poses a concern, as they are of a black-box nature, making it a challenge for clinicians to depend on their outcomes entirely. Thus, researchers are searching for alternative approaches, such as self-supervised learning, to lessen the reliance on labelled data while developing explainable AI techniques to enhance model predictions^3,4^. on the other hand, SL models often overfit to narrow labelled distributions, whereas SSL learns domain-invariant features through pretext tasks. DINOv2’s self-distillation inherently balances feature learning across classes.

While deep learning models have shown massive success in diagnosing medical images, much recent research focuses on conventional supervised learning. This reliance limits the use of these models in clinical settings where data is either scarce or challenging to annotate. On the other hand, Supervised models often overfit to narrow labelled distributions, whereas SSL learns domain-invariant features through pretext tasks. In addition, there is a need for models that achieve high accuracy and interpretability, enabling clinicians to trust their predictions**.** Current self-supervised approaches (e.g., DINOv1) partially address these issues but struggle with fine-grained medical feature extraction and integration into clinical workflows. Although some progress has been made with as self-supervised approaches like DINO, a direct comparison between these methods and traditional supervised models within specific medical contexts has not been thoroughly explored.

Our study aims to close this gap by comparing the evaluation of a self-supervised model(DINO) with a traditional supervised model across different medical imaging datasets, such as lung cancer, brain tumours, and leukaemia. In addition, we aim to evaluate the accuracy of these models by incorporating explainable AI methods, which will present significant insights helping in clinical decision-making. Our study aims to improve the performance of understanding the trade-offs between different strategic learning methods and contribute to developing more efficient and understandable medical imaging diagnosis algorithms.

This study aims to evaluate the result of self-supervised learning, explicitly using DINO V2^5^ in diagnosing medical images, and to compare its results with traditional supervised learning models. Given the challenges associated with labelling every sample in medical datasets, which is costly and time-consuming, this study proposes a self-supervised learning approach to extract valuable cancer-related features directly from the input data. Self-supervised learning^6^ has become more well-known for its capacity to create meaningful data representations from unlabeled data by creating pretexts, such as predicting characteristics of a masked or hidden region of a medical image based on the visible surrounding areas.

In our study, we have chosen the DINO method, which has demonstrated outstanding performance in image classification tasks like ImageNet while requiring significantly fewer computational resources than previous self-supervised approaches. By assessing DINO V2’s^7^ ability to extract meaningful features without extensive labeled datasets and comparing its performance across datasets, including lung cancer, brain tumors, and leukemia, with traditional supervised models, this research also aims to explore how explainable AI techniques can enhance model interpretability, thereby increasing clinician trust and facilitating the integration of these models into clinical workflows.

The rest of this paper is organised as follows: Sect. “Related work” summarises the related work—methods discussed in Sect. “Utilized dataset”. Section “Proposed work” discusses the dataset and the dataset processing. The proposed framework is discussed in Sect. “Results”. Section “Conclusion” presents the results and discussion.

## Related work

In this section, various researchers reported utilising ML and DL models for detecting multiple types of cancer^8,9^. For example, in^10^, Li et al. presented a hybrid feature extraction method for lung cancer classification. It combined Haralick and autoencoder features with the Grey level co-occurrence matrix (GLCM). By feeding these features into various supervised machine learning methods, such as SVM with Gaussian and Radial Basis Function (RBF), kernels achieved an accuracy of 99.56% and 99.35%, respectively, while SVM with a polynomial kernel, when utilising GLCM, reached an accuracy of 99.89%. Others in^11^ presented an early lung cancer detection approach using CT images embedded with a feature extraction technique. Karthiga et al. provided an Improved Naïve Bayes (I-NBC) classifier. The study applied the Accelerated Wrapper-based Binary Artificial Bee Colony (AWB-ABC) algorithm for feature extraction from the dataset (LUNA 16), achieving an accuracy of 98%.

Heidari et al.^12^ developed a deep learning-based method for lung cancer detection using chest CT images. They introduced a convolutional neural network (CNN) combined with a multiscale feature fusion approach to enhance the accuracy of early detection. Their model, leveraging Federated Learning and Blockchain systems, achieved a high testing accuracy of 99.69%, with F1 scores of 99.1% for detecting lung cancer and 98.9% for non-lung cancer. Bharathi et al.^13^ presented a hybrid attention deep learning network embedded with a heuristic algorithm for adaptive CT and PET image fusion in lung cancer detection, achieving an Accuracy of 97.41%. Ankışhan et al.^14^ presented an innovative non-invasive method for lung cancer detection utilising speech sound analysis. Their methodology employed Mel-frequency cepstral coefficients (MFCCS) and a convolutional neural network (CNN) to categorise speech signals from lung cancer patients and healthy subjects. Their model achieved an accuracy of 92.85% by extracting features through Voice Activity Detection and classifying them using a Graph Attention Network. Karthiga.

Others utilized Self-supervised learning (SSL) models for diagnosing. For example, Wang et al.^15^ introduced a pyramid self-supervised learning for histopathological image classification, Achieving an accuracy of 96.9%. This approach leverages multiscale feature extraction on the (BreaKHis) dataset. Agnes et al.^16^ developed the Wavelet U-Net +  + model for lung nodule segmentation using the ( LIDC-IDRI ) dataset. The model achieved performance with a mean Dice coefficient of 0.937% and an accuracy of 0.989%, particularly in detecting small and irregular nodules, by combining wavelet pooling with the U-Net +  + architecture. Gong et al. presented a method for detecting lung nodules resembling balls or dots. They employed the OTSU threshold, 3D region growth, and morphological operators as preprocessing filters for lung parenchyma segmentation^17^. Then, pulmonary nodules were detected using a multiscale 3d tensor filtering approach merged with local image feature analysis; after 442 2D and 3d features were retrieved, 19 features were chosen and fed into random forests for classification, and the nodule candidates were segmented and refined using a 3d level set method. In^18^, the authors utilized the LUNA16 Dataset and the ANODE09 Dataset. They achieved high rates of FP in CADe systems, which indicating widespread effect in clinical use. Table 1 summarizes the literature review with additional details. Applying self-supervised learning (SSL) techniques to biomedical data has gained popularity in recent years due to the difficulty in finding annotated datasets and the high expense of expert labeling. One significant trend is the use of generative and contrastive learning techniques to derive meaningful representations from unlabeled data, especially in fields like multi-omics and medical imaging. For multi-omics cancer classification, Hashim et al.^19^ presented Self-omics, a self-supervised system. Their approach creates robust embeddings that generalize across cancer kinds and modalities, especially in zero-shot and missing-modality circumstances, by combining contrastive and generative learning aims. SSL’s ability to tackle important issues in biomedical data analysis is demonstrated by this work. Similar to this, Coudray et al.^20^ created a self-supervised workflow to forecast unfavorable results from cutaneous squamous cell carcinoma (cSCC) histological images. Their method, which used whole-slide images of more than 700 patients, provided interpretability by finding histomorphological phenotypic clusters associated with prognosis in addition to achieving high predictive accuracy (c-index up to 0.84). Notably, their findings stratified risk in low-stage cancers (AJCC T2, BWH T2a), which are notorious for outcome heterogeneity, thereby addressing a significant therapeutic barrier. Zhou et al.^21^ presented Models Genesis, a unified self-supervised learning framework intended for 3D medical image analysis, extending the use of SSL to 3D imaging. Their method uses unlabeled 3D CT scans to learn general-purpose anatomical representations, in contrast to traditional transfer learning from natural images (e.g., ImageNet). To train encoder-decoder models that may transfer across organs, diseases, and modalities effectively, the system incorporates a number of pretext tasks, including non-linear intensity modulation, in-painting, and local pixel shuffling. Across a variety of segmentation and classification tasks, their results demonstrated consistent gains over training-from-scratch 3D models and supervised 2D transfer learning. Nevertheless, a significant gap still exists in the literature: few research directly compare SSL and supervised learning techniques on the same biomedical tasks and datasets. It is challenging to determine the actual added value of SSL in clinical settings due to the absence of benchmarking. Additionally, the majority of SSL applications prioritize representation quality over interpretability and downstream job performance, both of which are crucial in healthcare settings.

**Table 1:** Literature review summarization.

References	Utilized data	Techniques	Performance	Advantages	Limitations
^12^	Chest CT images from multiple institutions	Federated Learning, Blockchain Systems	Acc = 99.69%	Ensures data privacy and security improves detection accuracy, enables collaboration without data sharing	Requires significant computational resources Not considering potential data heterogeneity issues
^13^	PET and CT images of 90 patients from an online dataset	CNN for image fusion MIV-CapSA for hyperparameter optimisation, TransUnet3 + for segmentation, HADN for cancer node detection	Acc = 97.41%	Early detection of lung cancer Enhancing image quality through fusion, effective segmentation Provide high accuracy in node detection	Complexity in model training Require high-quality images, potential challenges in generalizing
^14^	Speech recordings Features: 35 time and frequency features reduced to 17–19 significant features	Feature Extraction: Voice Activity Detection Dimension Reduction: Model: Graph Attention Network with fine-tuning for classification	Accuracy: 92.85%	High Accuracy Feature Relevance Practical Application	Speech Variability Requires sophisticated models and careful tuning due to the intrinsic complexity of speech data
^10^	Lung cancer images	image resizing, data augmentation, grid search, random search, k-fold cross-validation, feature extraction (Haralick, GLCM, Autoencoder)	Acc = 99.89%	Improved accuracy and generalization through data augmentation and robust validation, hyperparameter optimization	Computational complexity potential overfitting
^15^	Large-scale unlabeled histopathological images	Self-supervised learning Pyramid-based Local Wavelet Transformer (PLWT) Wavelet for downsampling in multi-head attention Local Squeeze-and-Excitation (Local SE) module	Acc = 96.9%	Reduced need for labelled data Richer feature extraction Less information loss during feature transmission Improved generalization performance	Complexity in model design and implementation Potential computational and storage demands due to advanced techniques
^16^	The study utilized the LIDC-IDRI dataset	Wavelet U-Net + +	Acc = 98.9 ± 0.08	Improved lung nodule segmentation accuracy, especially for small and irregular nodules	Increase the model’s complexity
^18^	The LUNA16 Dataset and the ANODE09 Dataset	SVM	Sensitivity = 79.3%	High Sensitivity in Nodule Detection	High rates of FP in CADe systems indicate an effect that is widespread in clinical use
^19^	Multi-omics cancer datasets	Self-Supervised Learning using generative and contrastive objectives	Acc	Enables learning from unlabeled omics data, generalizes to unknown cancer kinds (zero-shot learning)	Lacks direct comparison with traditional supervised models in all tasks
^20^	Histopathology Images of cSCC	Self-supervised learning Histomorphological Phenotype Learning (HPL)	c-index , AUC	Enables unlabeled early risk categorization, interpretable phenotypic groupings, and potent low-stage tumor performance	Model complexity requires WSI preprocessing and computational resources
^21^	Unlabeled 3D CT scans, X-rays (ChestX-ray8), Ultrasound, MRI	Unified self-supervised learning framework (Models Genesis	AUC, LOU	Exploits 3D anatomical structure, no labels required, robust across tasks	Complex architecture; limited public 3D benchmarks
^22^	LIDC-IDRI Dataset,NIH Chest X-ray Dataset	Using a deep pyramidal residual network	Acc = 97.88%	Using CLAHE enhances image quality and lung image segmentation with the Honey Badger Algorithm	Time-intensive for each image to accurately detect and segment lung regions
^23^	IQ-OTH/NCCD Dataset, Chest CT-Scan Dataset	GoogLeNet-AL	Acc = 98.74%	Efficient multiscale feature extraction	Deploying GoogLeNet-AL in clinical settings may be limited by its computational needs and dataset quality

## Background and methods

### Self-supervised learning for medical images

In modern healthcare, medical imaging technologies have become indispensable in decision-making, diagnosis, and treatment planning. As a result of the growth of technologies, their significance in healthcare systems has become increasingly apparent. However, this growth also brings a fresh obstacle: the disparity gap between the increasing number of medical images and the available radiologists. This discrepancy has resulted in a notable increase in the workload of radiologists, requiring them to operate at a very rapid speed, analysing an image every 3–4 s to fulfil clinical needs^24^. The large amount of work has led to more mistakes in diagnosing, highlighting the need for automated systems to enhance diagnostic efficiency. Deep learning models have been used as a solution for this scenario. These models have shown that they can achieve expert-level accuracy in specific imaging tasks like X-rays^25^ and CT scans^11^. Yet, these models heavily depend on supervised learning, necessitating accurately annotated datasets. Because of the requirement for specific knowledge in annotation, producing these datasets is expensive and time-consuming, restricting the advancement of efficient models for different clinical purposes. This is the starting point for researchers to investigate new methods to tackle these challenges. Self-supervised learning (SSL) is a modern medical image analysis approach. This approach has the ability to extract valuable information from huge amounts of unlabeled data using tasks guide the model to learn rich representations can be used to improve performance as well as decreasing manual cost annotations, the time and resources required for developing the model.

In this context, self-supervised learning has been utilized in scientific research related to the “Pyramid-based Local Wavelet Transformer” (PLWT) model for analysing histopathology images. This model relies on pre-training with large quantities of unlabeled images, which aids in extracting rich image representations that enhance the model’s performance in subsequent tasks. In applying COVID-19 classification and segmentation, a few works were devoted to the severity assessment of COVID-19. For example, Song et al*.*^26^ propose a novel self-supervised deep learning method for automated segmentation of COVID-19 infection lesions and assessing the severity of infection in 3d CT images by jointly performing lung lobe segmentation and multi-instance classification. Bai et al.^27^ proposed self-supervised features by predicting anatomical positions for cardiac MR image segmentation. In^28^, Taleb et al. proposed a multimodal puzzle self-supervised method, facilitating rich representation learning from multiple image modalities. Additionally, as in a few narrative reviews^29,30^, the most suitable strategies and best practices for medical images have not been sufficiently investigated.

### Detection transformer with improved denoising anchor

The detection transformer with an improved denoising anchor (DINO) is a self-distillation (self-supervised model) that represents a transformative model that integrates the data-driven methodology to improve the efficiency and interpretation of the model. It was integrated with a Vit transformer to enhance the overall classification. Performance^7^ The Vision Transformer (Vit)^31^ offers a transparent and interpretable deep learning method. Unlike many traditional models, ViT emphasizes the spatial arrangement and relationships between objects within an image. It leverages the concept of “attention” by integrating information from various “attention heads, each of which concentrates on different features of the image^32^. This approach allows for visualizing attention patterns, enhancing the model’s transparency. As a result, ViTs are gaining popularity in medical research.

This combination aims to detect recurring image patterns without relying on image labels.

**Fig. 1:**
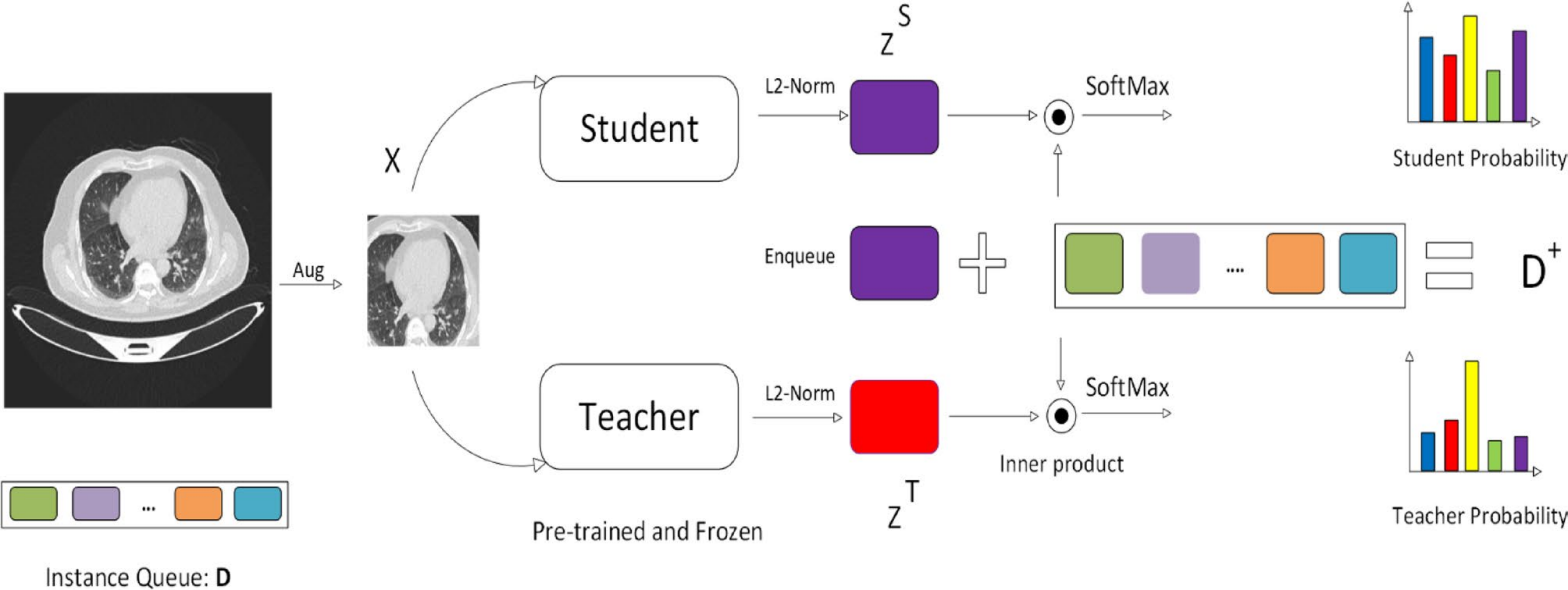
Self-distillation with no labels.

DINO is explained with a single pair of image views (× 1, × 2) for simplicity. The model applies two random transformations to the same input image, which are then fed into the student and teacher networks. While the networks share the same architecture, their parameters differ. The output from the teacher network is centered using the mean computed across the batch. Both networks produce K feature vectors, normalized using a temperature-scaled SoftMax. The similarity between these outputs is measured with a cross-entropy loss. A stop-gradient (sg) operation is applied to the teacher network, allowing gradients to flow only through the student network. The teacher’s parameters are updated using an exponential moving average (EMA), as shown in Fig. 1 of the student’s parameters.

1 $$P(y = j|x) = \frac{{e^{{x^{T} w_{j} }} }}{{\sum\limits_{k} {e^{{x^{T} wk}} } }}$$

#### Dino & traditional knowledge distillation

In DINO, both the student and teacher models are updated, whereas in traditional knowledge distillation, only the student model is trained, with the teacher model remaining static. DINO requires the student and teacher networks to have identical architectures to facilitate effective weight updates. In contrast, traditional distillation allows for flexibility in model architectures, enabling the student and teacher models to differ in structure. Traditional Knowledge Distillation utilizes KL Divergence loss, which measures how the student model’s output distribution Q diverges from the teacher’s output distribution P, as shown in Eq. 2.

2 $$KL(P||Q) = \mathop \sum \limits_{i} p\left( i \right) log\left( {\frac{P\left( i \right)}{{Q\left( i \right)}} } \right)$$

2-DINO: Focuses on contrastive learning, emphasizing the similarity of feature representations. The Cross-Entropy loss in DINO is calculated using the distribution of the positive pair, specifically the teacher’s representation P of the student’s image Q:

3 $$Cross\;Entropy\;\left( {P,Q} \right) = - \mathop \sum \limits_{i} p\left( i \right)\log \left( {Q\left( i \right)} \right)$$

### Dino V2

The architecture of DINO V1 is modified in various ways by DINO V2^33^, which adds new image-level and patch-level objectives. The image-level goal is carried over from DINO V1, which computes the loss solely using the features from the class token [CLS]. Using the patch feature outputs of ViT, the DINO V2 architecture presents a patch-level objective. Figure 1 More specifically, all instructor patches are left intact, but a mask taints some student input patches before being fed into the Transformer. Subsequently, the image-level loss is supplemented with a cross-entropy loss between the matching patch features of the teacher and student.

**Fig. 2:**
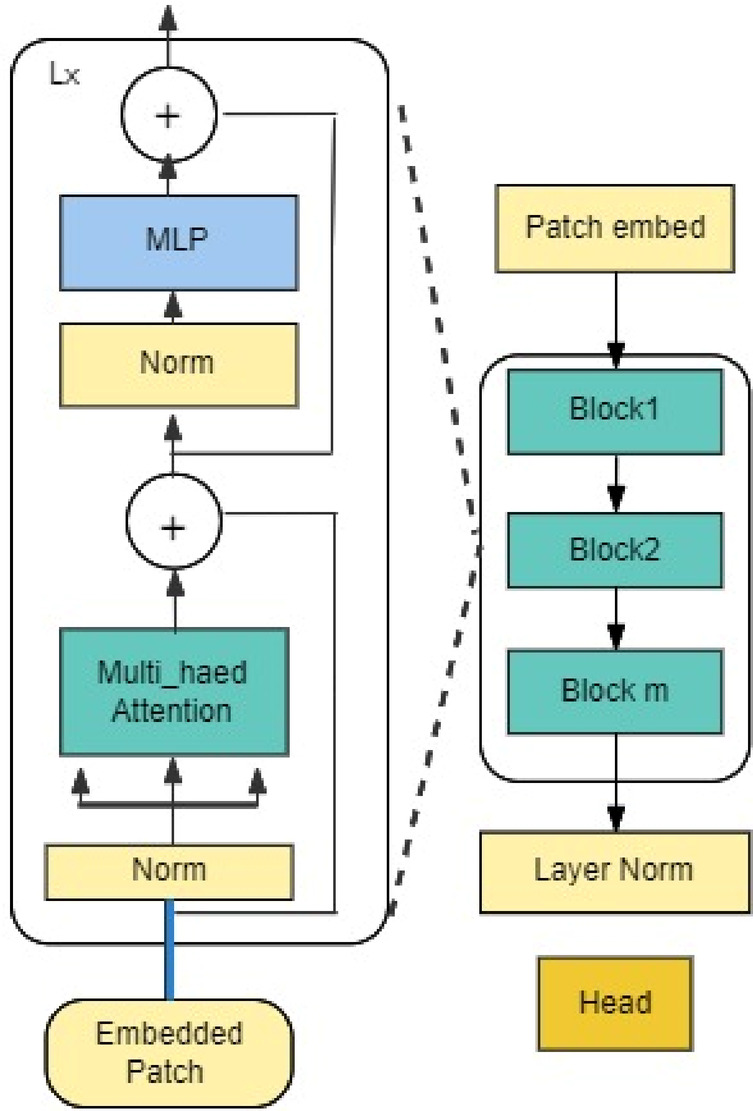
Dino v2 architecture.

The DINO V2^5^ head is attached to the backbone’s end and customized for the particular task. When classifying images, for instance, the head might be made up of a linear classification layer that utilizes the vector representation the backbone produces to categorize the image into distinct groups. The head will comprise a detection network that locates and classifies items in the image using the vector representation if object detection is performed. Figure 2 shows DINO V2.

### Barlow twinss

Is an approach to self-supervised learning that uses the redundancy reduction principle, which was first proposed in neuroscience. The objective function measures the cross-correlation matrix between the embeddings of two identical networks fed with distorted versions of a batch of samples and tries to make this matrix close to the identity. This causes the embedding vectors of a distorted version of a sample to be similar while minimizing the redundancy between the components of these vectors.

### Bootstrap your own latent (BYOL)

A new approach to self-supervised image representation learning. BYOL is built on two neural networks, known as the online and target networks, that interact and learn from one another. The online network is trained to predict the target network representation of an image under a different augmented view using an augmented view of the picture. At the same time, the target network is updated with the online network’s slow-moving average. While state-of-the-art approaches require negative pairs to function, BYOL creates a new state-of-the-art without them.

### Explanation artificial intelligence

In medical domains, especially cancer diagnosis tasks, ensuring the transparency and trustworthiness of model predictions is critical because these decisions directly affect patient outcomes. The black-box nature of deep learning models poses a significant challenge, making the incorporation of explainable AI (XAI) techniques essential to building confidence and trust in model predictions among clinicians^34^. To explain the decisions made by Vision Transformer models, A technique called Vit-CX: Causal Explanation of Vision Transformers “Vit-CX) offers an approach for transformer architecture, providing causal explanations beyond simple correlation-based interpretations. It is designed to explain the decisions of vision transformers^35^. The Vit-CX method analyzes causal relationships between input tokens and model output predictions. This approach matches DINO V2, the self-supervised learning Vision Transformer, in analysing lung, brain, and leukaemia cancer data. This method computes token-level importance scores and highlights the most causally influential parts of the input image for the model’s decision^36^.

Vit-CX explains how Vision Transformers (ViTs) make predictions by focusing on the important parts of an image by taking the class prediction, calculating the gradients to see how much each image patch influences the result, and combining that with the model’s attention to highlight key areas. Instead of just using attention weights, Vit-CX looks at the patch embeddings (the content of the image patches) to create masks that show which parts of the image are most important. It then calculates the causal impact of each patch to see how changes in the image affect the prediction and corrects any biases to ensure a fair explanation. This results in a saliency map showing which parts of the image were critical to Vit’s decision^35^. Vit-CX is applied to the DINO V2 model after processing the medical images. The resulting explanations are visualised as heat maps like the original images, indicating the regions of high causal importance. These visualizations improve the diagnostic process overall by helping medical professionals to evaluate and understand the AI systems in medical fields and also help in the interpretation of models^37^. This approach improves the power of self-supervised learning through DINO V2 while maintaining a high standard of interpretability and trustworthiness in the cancer diagnosis support system.

## Utilized dataset

In this study, we utilize three different datasets, each corresponding to a specific type of cancer: lung, brain, and leukaemia. These datasets encompass various medical imaging data, providing a diverse and comprehensive representation of each cancer type.

### Lung cancer datasets

**Fig. 3:**
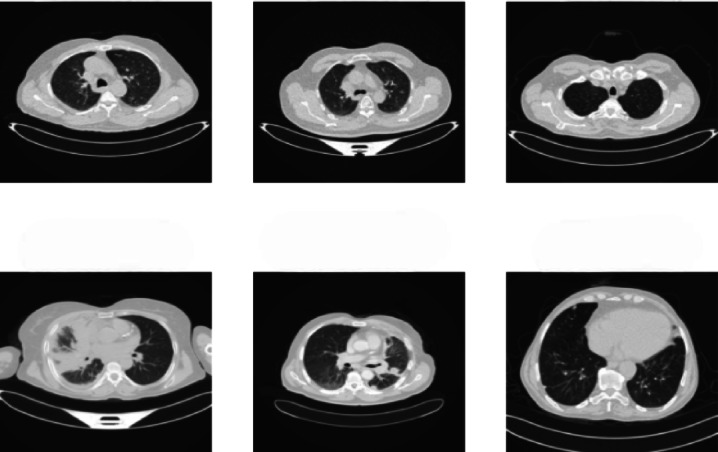
Lung cancer dataset.

The lung cancer dataset^38^ from the Iraq-Oncology Teaching Hospital/National Centre for Cancer Diseases (IQ-OTH/NCCD) was gathered over three months in the fall of 2019 at the aforementioned speciality hospitals. It comprises both healthy subjects’ and patients’ CT scans that have been diagnosed with lung cancer at various stages. In these two centres, radiologists and oncologists marked IQ-OTH/NCCD slides. The dataset includes one thousand one hundred ninety pictures representing CT scan slices of 110 instances (Fig. 3). The three groups of these cases are malignant, benign, and routine. Of these, 40 cases have been classified as malignant, 15 as benign, and 55 as usual. Figure 3 shows the lung cancer images.

### Brain tumour dataset

**Fig. 4:**
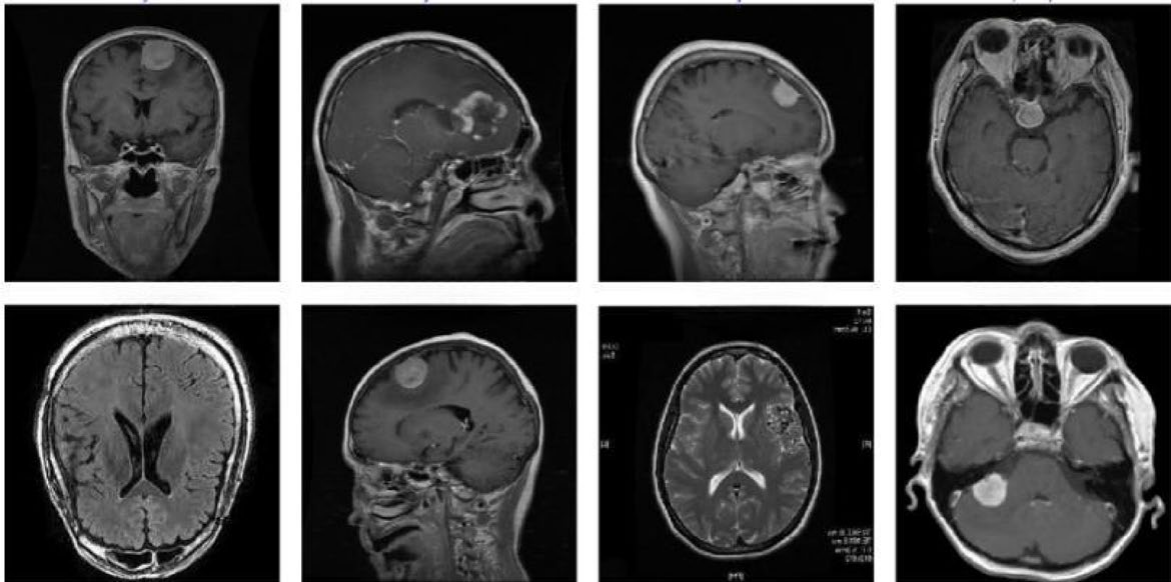
Brain tumour dataset.

This dataset^39^ comprises 7,023 MRI images of the human brain, divided into four categories: pituitary, glioma, meningioma, and no tumour. The data is sourced from Figshare, SARTAJ, and Br35H. Due to the integration of these diverse sources, the image sizes vary, ranging from 512 × 512 to 219 × 234. The dataset Fig. 4 is organized into two folders: a training folder containing 5,712 images and a test folder containing 1311 images.

### Acute lymphoblastic leukaemia dataset

**Fig. 5:**
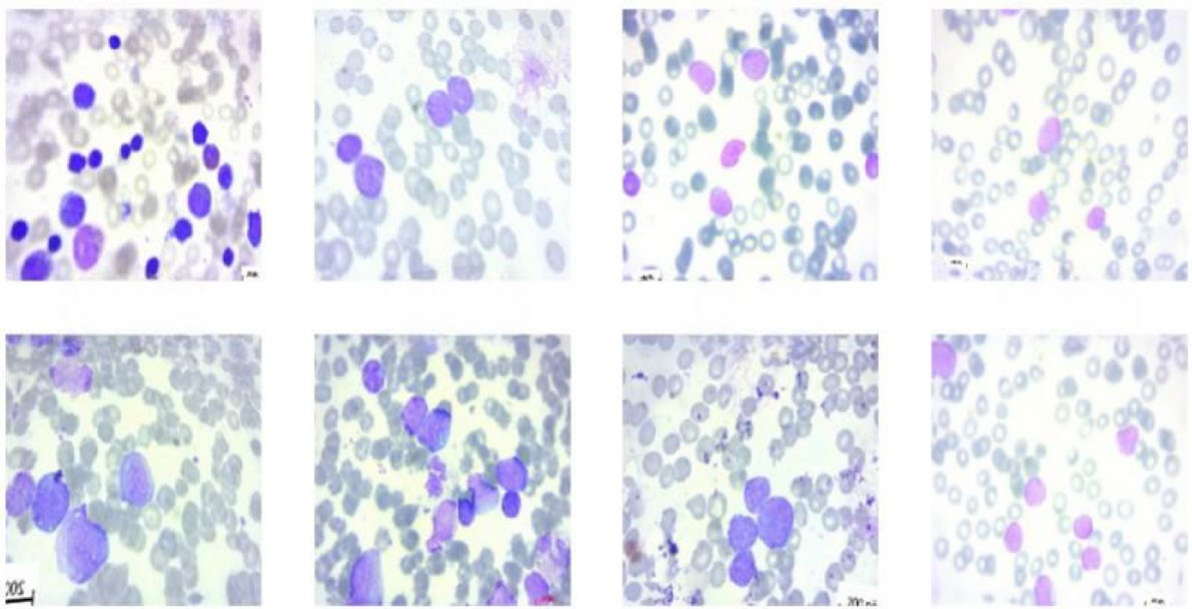
Acute lymphoblastic leukaemia dataset.

The Taleqani Hospital’s (Tehran, Iran) bone marrow laboratory produced the images for this dataset^40^. 3256 PBS pictures from 89 suspected ALL patients were included in this dataset—skilled laboratory personnel prepared and stained the blood samples. Benevolent and malignant classes are distinguished in this dataset, Fig. 5. Hematogones comprise the former category, whereas the ALL group includes Early Pre-B, Pre-B, and Pro-B ALL malignant lymphoblast subtypes.

### Eye disease retinal images

**Fig. 6:**
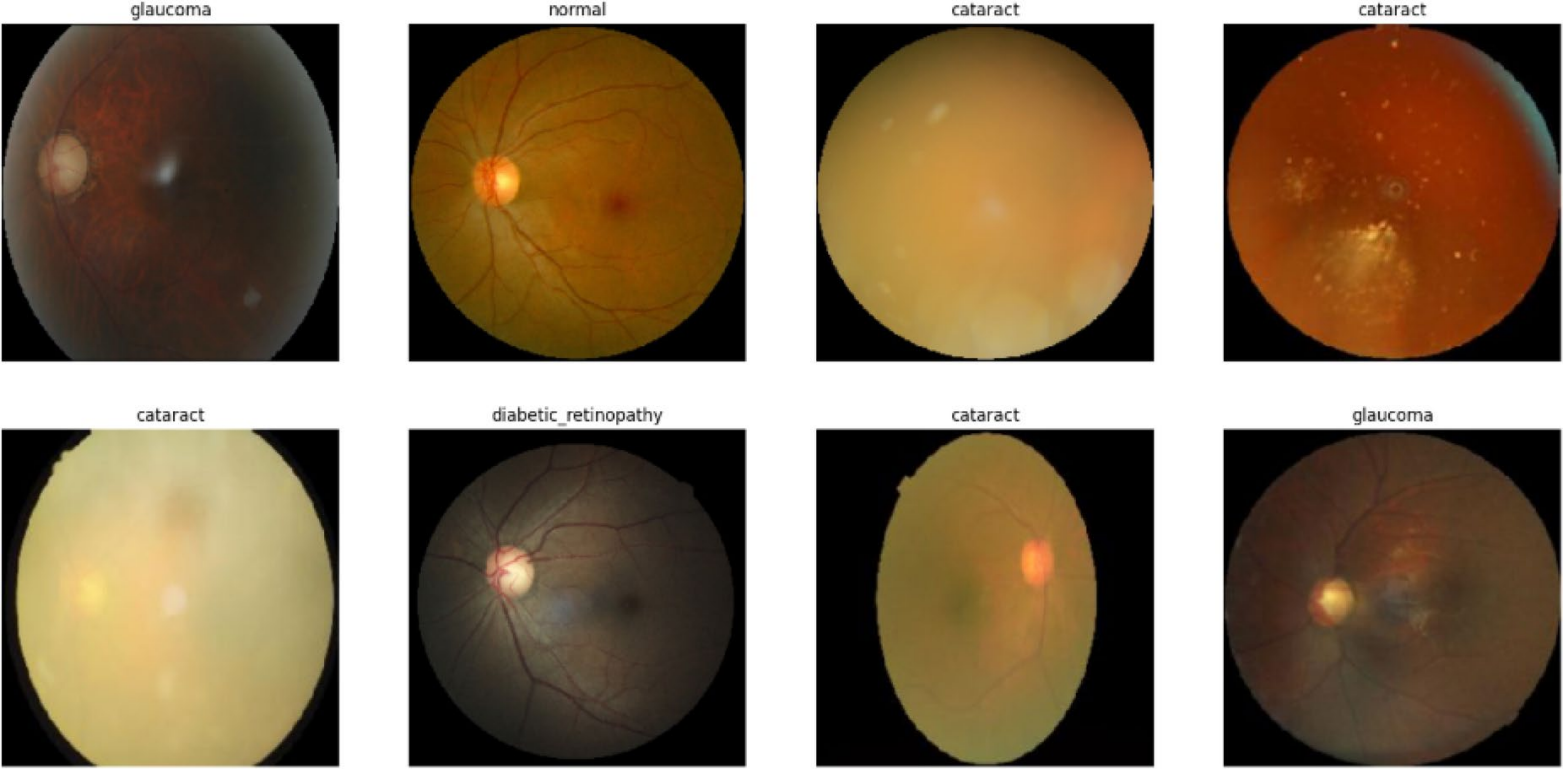
Eye disease retinal images dataset.

This dataset consists of retinal images categorized into four distinct classes: Normal, Diabetic Retinopathy, Cataract, and Glaucoma, as shown in Fig. 6, where each class has approximately 1000 images. These images are collected from various sources like IDRID (Indian Diabetic Retinopathy Image Dataset), Ocular recognition, and HRF(High-Resolution Fundus) Fig. 6 shows the Eye Disease Retinal Images dataset.

### Data preprocessing

#### Data augmentation

In this study, we apply several data augmentation techniques to enhance the model’s robustness, prevent overfitting, balance class distributions, and introduce variability in the training data. The essential augmentations used are:

- *RandomHorizontalFlip*: This augmentation flips images horizontally with a given probability. It helps the model become invariant to the orientation of the input images, ensuring it can accurately identify features regardless of their left–right orientation.
- *CenterCrop*: Centre cropping involves cropping the image’s central region to a specific size. This technique focuses on the most relevant part of the image, removing unnecessary background noise, which can improve the model’s focus on critical features.
- *RandomResizedCrop*: Using this method, the image is first resized to a desired size after a random section has been cropped. This helps the model learn different image sizes and generalise better.

**Table:** 

RandomHorizontalFlip	Size	256 × 256 pixels (standard for DINOv2)
	Scale	[0.6, 1.0]
	Ratio	[0.8, 1.2]
CenterCrop	Size	224 × 224 pixels for validation
Normalization	Used ImageNet defaults	mean = [0.485, 0.456, 0.406], std = [0.229, 0.224, 0.225]

To guarantee the performance of our models, we did not use colour space transformations, kernel filters, or mixing between images. Using geometric transformations, we maintained the original image’s integrity and introduced the necessary variations for reliable model training.

#### Image normalization and resizing

To ensure consistency and increase the speed of model training, datasets undergo image normalization and resizing.

**Fig. 7:**
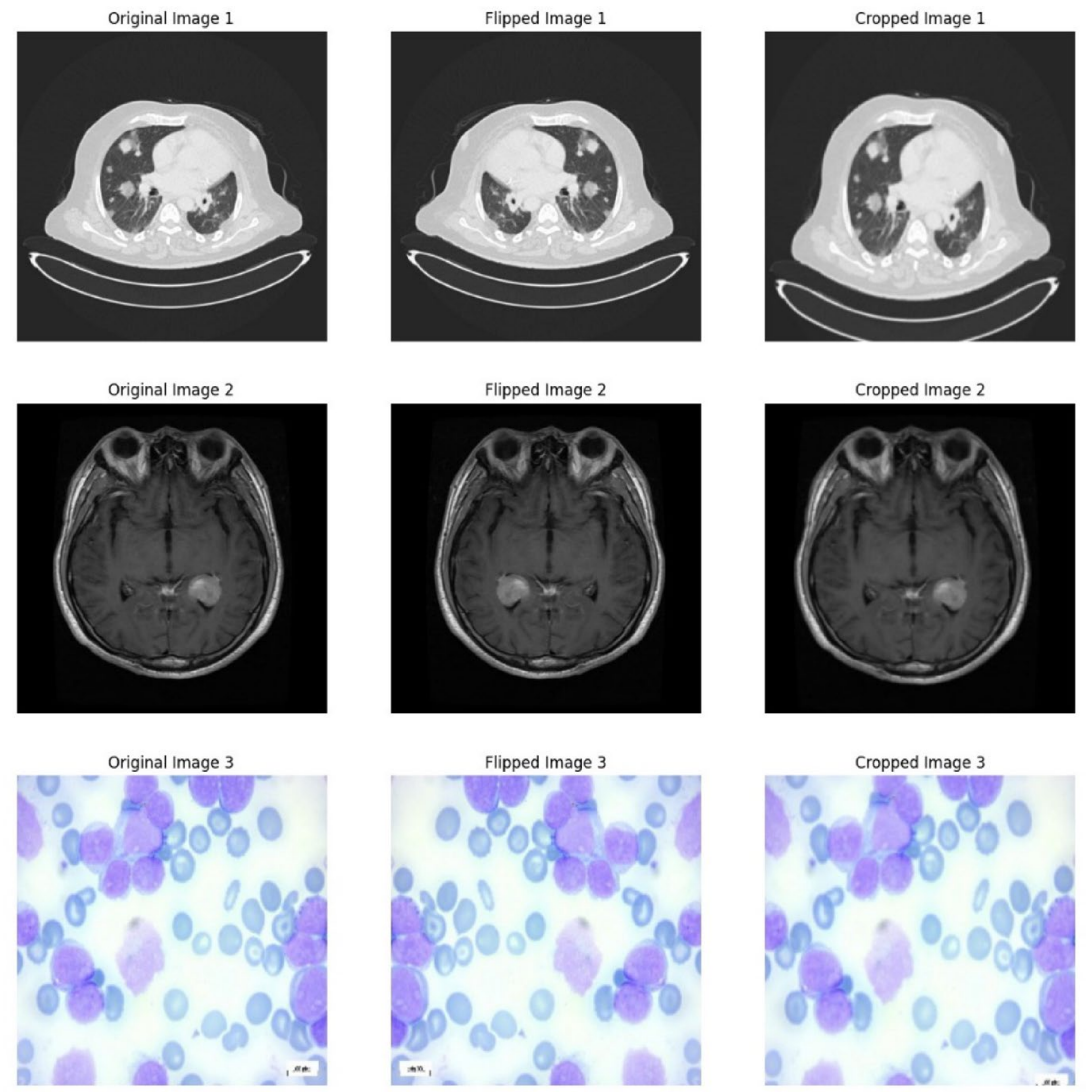
Data preprocessing examples.

- *Image resizing*: To ensure compatibility with our models, we assign all images to a fixed size. This stage is essential for preserving the integrity of the spatial features.
- *Image normalization*: This process alters the range of pixel intensity values during image processing. Its primary purpose is to transform an image into a spectrum of pixels that are more familiar or normal to the senses. It prevents the model’s learning from being skewered by fluctuating pixel intensities, which helps stabilize and accelerate the training process. Figure 7 shows the examples of the images after the preprocessing steps.

## Proposed work

### Our proposed work has three main steps as follows

#### Model development

**Fig. 8:**
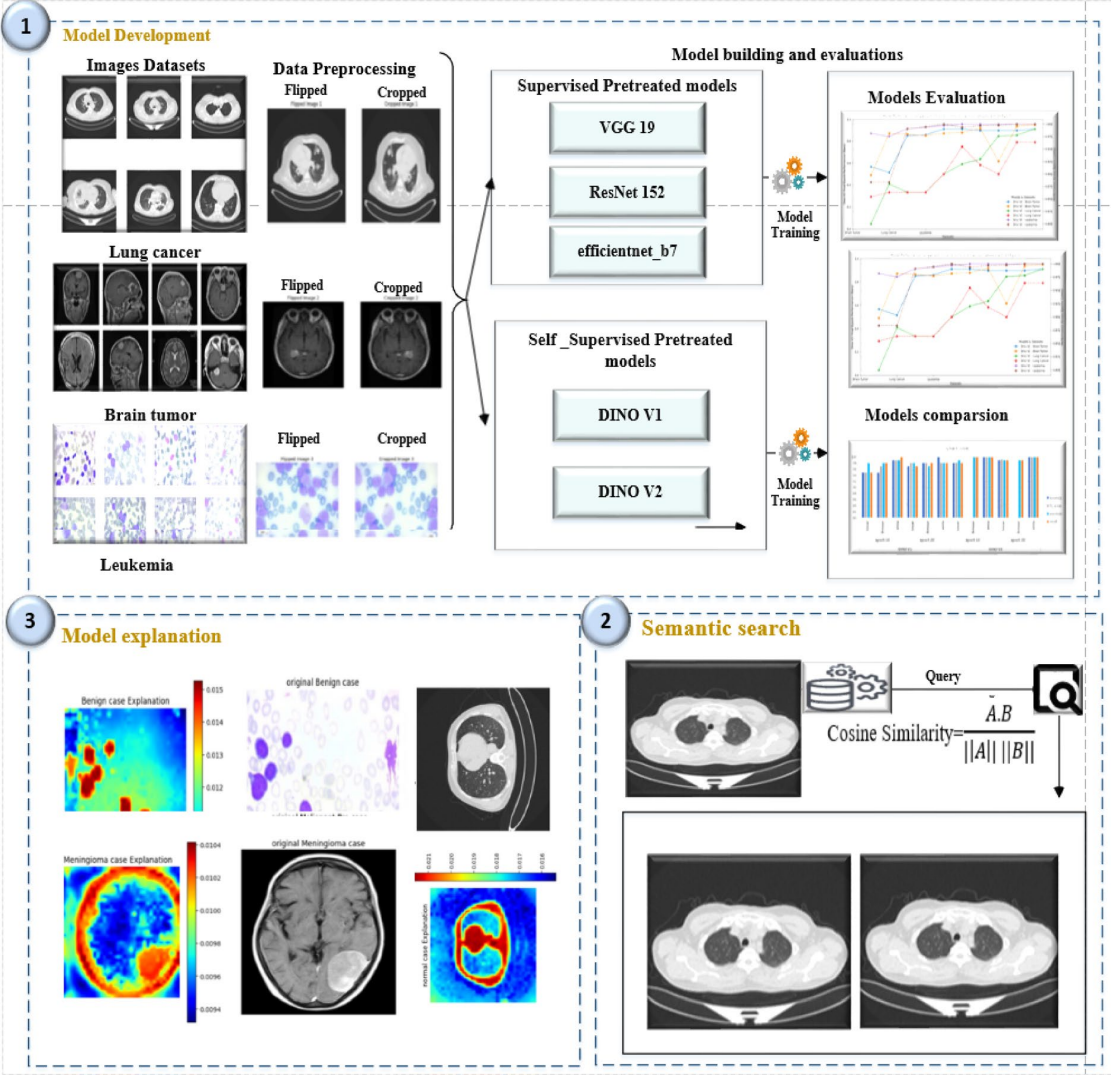
Proposed framework.

This step includes data aggregation, data processing, and designing and building the predictive model, which will be used further. In this paper, our hypothesis is to measure the effect of using fine-tuning for self-supervised and supervised models and the impact on medical image diagnosis, which has demonstrated significant power in recent years. As shown in Fig. 8, we chose to work with medical data to ensure that these models had no pre-trained related data, thereby measuring their capability for generalization and learning features from a different domain. This approach allowed us to determine which model would perform better than others. Two preprocessing steps were applied to the utilized dataset: data augmentation and data normalization. Data augmentation occurred using the following functions (RandomHorizontalFlip, CenterCrop, and random resized crop). Then, all data was resized and normalized to the same image size to speed up the training and maintain image consistency.

We compared it with DINOv2, a foundation model pre-trained on large quantities of data in a self-supervised manner to learn robust visual features without supervision, which has shown significant results. To make a fair comparison, we compare DINO v2 with DINO v1, BYOL and Barlow Twins and popular supervised models, including VGG, ResNet, and Efficient Net architectures. Furthermore, we aim to maintain a robust learning pipeline for our models. We choose not to perform any optimization for the models’ hyperparameters (learning rate set to be constant = 1e^−5^, training epochs = (10, 20)). These settings are repeated for training for all models to ensure that all models take the needed time to learn from the data and that the gradient accumulation and warmup ratio are constant for all the models. To ensure comparable learning capabilities, we chose our supervised models to be of the maximum size as follows.

- Dinov2 model’s size = 86,8 M parameters
- VGG19 model’s size = 144 M parameters
- ResNet-152 model’s size = 60,3 M parameters
- Efficentnet-B7 model’s size = 66 M parameters

All the parameters of the backbones for the models are frozen, and a classifier head forces the models to learn from the final embeddings. To overcome the mismatched sizes from the pre-trained model and ensure the fine-tuning of a pre-trained model on a new dataset with different input sizes (medical image data), HF with Pytorch was utilized to ignore mismatched sizes to allow the model to adjust its layers to accommodate size differences without throwing an error.

#### Semantic search

After completing the model training process, the embedding derived from the model provides an efficient representation of the semantic meaning of the input data. These embeddings encapsulate the relationships in the input data and permit robust semantic search capabilities^41^. Our paper used the Quadrant database to save the embedding from the developed model. Quadrant offers storage that can efficiently manage embedding. It also ensures the security of the embedding developed during training, enabling reliable access to semantic information for further processing. With scalability, the quadrant also proved its scalability ability for robust semantic search^42^.

In our study, semantic search leverages embedding to deliver search results for user queries. The semantic search was performed by measuring the cosine similarity between the user query and the information embedding. The semantic search could retrieve the semantically relevant images by comparing the indexed search via model embedding and the user query.

#### Model explanation

In medical image classification, explanation is essential for augmenting model transparency, offering insights that facilitate the model. This phase enhances the interpretability and transparency of the model’s decisions and predictions. Our article provides a representation that emphasizes the most relevant components in the model’s decision-making process. Our work used the ViT CX explanation for the DINO v2 vision transformer model for three cancer classification tasks. The findings provide insights into DINO v2’s classification methodology, emphasizing critical criteria for differentiating cancer kinds and stages. Due to the delicate nature of cancer diagnosis, comprehending the model’s decision-making process is essential for fostering confidence in AI-assisted medical picture analysis^43^. Analysing these heatmaps in conjunction with the original photos enhances our comprehension of the model’s focus, facilitates the identification of clinically relevant characteristics, and ensures the model’s dependability in this vital healthcare application.

## Results

### Experimental setup

All experiments were conducted on 4 × NVIDIA V100 GPUs (32GB VRAM) with the following considerations: (1) DINOv2 training used a batch size of 32 (Sect. “Proposed work”) to balance memory constraints with gradient stability during self-distillation; (2) input resolutions were standardized to 256 × 256 pixels to preserve diagnostic features while maintaining throughput; and (3) ViT-CX explainability operations added < 15% inference latency. To evaluate the performance of our proposed model, we employed various metrics, including accuracy, precision, recall, and F1 score. Results were computed using the training data, while the generalization performance was assessed using the testing data. First we use accuacry which accuracy is calculated as the proportion of correctly classified cases out of the total cases, percison: that the percentage of cases of the positive class that are accurately classified. Recall: which is is determined by the ratio of correctly classified positive records to the total number of classifications within the class and the F1_scocre that represent, the harmonic mean of precision and recall, is a robust evaluation measure for imbalanced data.

4 $$\text{Accuarcy }= \frac{tp+tn}{tp+fp+tn+fn}$$

5 $$\text{Percsion }= \frac{tn}{tn+fp}$$

6 $$\text{Recall }= \frac{tn}{tn+fn}$$

7 $$\text{F}1\_\text{score }= \frac{2(P*R)}{P+R}$$

### Supervised models

**Fig. 9:**
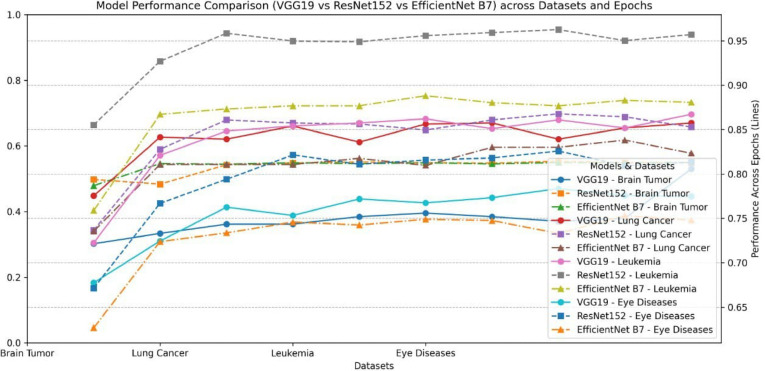
Comparison between all pre-trained models.

In this section, we evaluate the performance of pre-trained models, including VGG19, ResNet15, and EfficientNet_B7, in medical image classification across various datasets, including lung cancer, brain tumours, and leukaemia. From the results in Table 2, we can observe the following:

1. For lung cancer prediction, VGG performs best, with an accuracy of 0.857 and an F1 score of 0.8301. This is followed by ResNet, which achieved a similar performance with an accuracy of 0.8529 and an F1 score of 0.83308. The worst result is from the Efficient Net. This could be attributed to the structure of the VGG model, which makes it easier to train and optimize for medical image tasks than the Efficient Net.
2. In predicting brain tumours, the worst performance is obtained from the VGG model, achieving an accuracy of 0.7531. Utilising ResNet enhances the overall performance by 5% over VGG. Similarly, Efficient Net attained an accuracy of 0.8671 and an F1 score of 0.8913.
3. Regarding the results with the leukaemia dataset, ResNet demonstrates superior performance with an accuracy of 0.957 and an F1 score of 0.976. It is followed by the Efficient Net model, which is achieved. Figures 8 and 9 show a comparison between all supervised learning models.

**Table 2:** Results of the supervised learning.

Dataset	Model	Accuracy	F1_score	Precision	Recall
Lung cancer	vgg19	0.8575	0.8301	0.8287	0.8318
	resnet152	0.8529	0.8308	0.8252	0.8523
	efficientnet_b7	0.8233	0.7795	0.7979	0.8169
Brain tumor	vgg19	0.7531	0.7388	0.6723	0.8199
	resnet152	0.8119	0.7388	0.6723	0.8199
	efficientnet_b7	0.8099	0.7383	0.6721	0.8189
Leukemia	vgg19	0.8671	0.8913	0.8984	0.8982
	resnet152	0.9570	0.9760	0.9760	0.9760
	efficientnet_b7	0.8806	0.9195	0.9220	0.9213
Retina	vgg19	.83	.837	.8410	.8328
	resnet152	.8423	.8420	.435	.8423
	efficientnet_b7	.8139	.8160	.258	.8139

While the current results demonstrate the effectiveness of supervised learning in medical diagnosis, various limitations may hinder the generalization ability of traditional supervised learning, which could be addressed by utilizing self-supervised learning. These limitations can be summarized as follows:

1. Supervised learning models may struggle with imbalanced data, leading to a bias towards the majority class and hindering their generalization ability. In contrast, self-supervised learning can learn more balanced representations, avoiding bias towards the majority class.
2. Supervised learning can learn from labelled data but may face several challenges in generalizing to unseen data. In contrast, self-supervised learning can extract more transferable representations of features, resulting in a model with better generalization.
3. One of the main challenges in medical data is the labeling process, which may not commonly exist. This challenge hinders its ability to benefit from this data. Self-supervised learning can capture significant features that could be utilized in various AI tasks.
4. Medical images such as MRI, histopathology, and CT scans usually include complex details requiring special feature extraction to identify objects. Self-supervised learning can extract significant information automatically from such images.

**Fig. 10:**
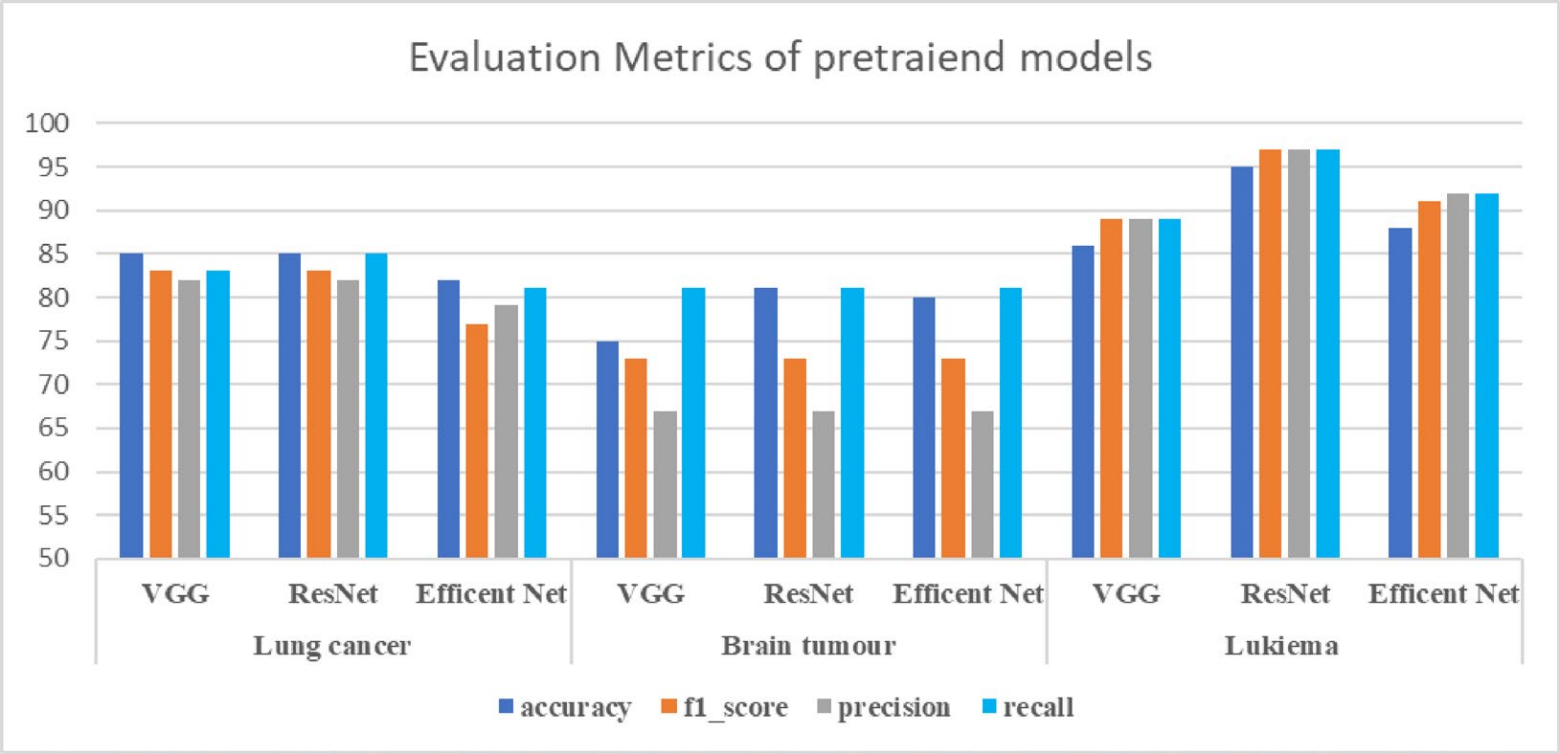
Comparison between all supervised learning in terms of all evaluation metrics.

These challenges lay the groundwork for utilizing self-supervised learning in medical diagnosis. The subsequent section will concentrate on the abilities of self-supervised learning, as exemplified by DINO V1 and V2 and its ability to provide promising performance compared to supervised learning. Figures 9 and 10 show a comparison between all utilized models

### Dino V2 model

This section analyzes the performance of Self-supervised learning models exemplified in DINO V1 and V2 across various datasets in medical image classification.

After training for ten epochs, DINO V1 achieved promising performance in several evaluation metrics. For example, the performance in brain tumour tasks. DINO V1 achieves 97.8%, 98.2%, and 98% in accuracy, precision, recall, and F1-score. This result demonstrates the ability of DINO to provide more accurate classification compared to supervised learning. The same applies to leukaemia and brain tumour tasks, as shown in Table 3.

**Table 3:** Results of the self-supervised learning.

	Dataset	Dataset	Accuracy	F1_score	Precision	Recall
DINO V1	10 Epochs	Lung cancer	0.9532	0.97	0.96	0.97
		Brain tumor	0.97.8	0.98	98.2	0.981
		Leukemia	0.982	0.976	0.99	0.973
	20 epochs	Lung cancer	0.9	0.99	0.98	0.97
		Brain tumor	0.99	0.99	1.00	1.00
		Leukaemia	1.00	1.00	1.00	0.99
DINO V2	10 Epochs	Lung cancer	0.9836	0.9621	0.95	0.95
		Brain tumor	0.9986	0.9986	1.00	1.00
		Leukaemia	1.00	1.00	1.00	1.00
	20 Epochs	Lung cancer	0.99	0.97	0.97	0.97
		Brain tumor	0.994	0.994	0.99	0.99
		Leukaemia	1.00	1.00	1.00	1.00

These outcomes highlight the ability of DINO v1 to provide promising performance, and the precision and recall affirm its robustness in capturing significant patterns.

Upon increasing the number of epochs to 20, the overall performance was enhanced by approximately 1–3%. For example, for lung cancer, the developed model achieves accuracy, precision, and recall of 99%, 97%, 97%, and 98%, which outperforms the performance with ten epochs. Similarly, the model achieves accuracy for lung cancer detection and F1-score of 99%, 99%, and 99%. The balance between precision and recall results ensures the DINO’s robustness in providing stable predictions across various classes.

The model can extract the significant features affecting the overall evaluation metrics, including precision, recall, and F1-score. These results demonstrate the model’s ability to handle different datasets with minimal errors. The promising results from DINO V1 and DINO V2 shed light on the importance of utilizing data-centric models, which could enhance decision-making. Validating these results in various datasets prepares the way for integration in the healthcare domain.

**Fig. 11:**
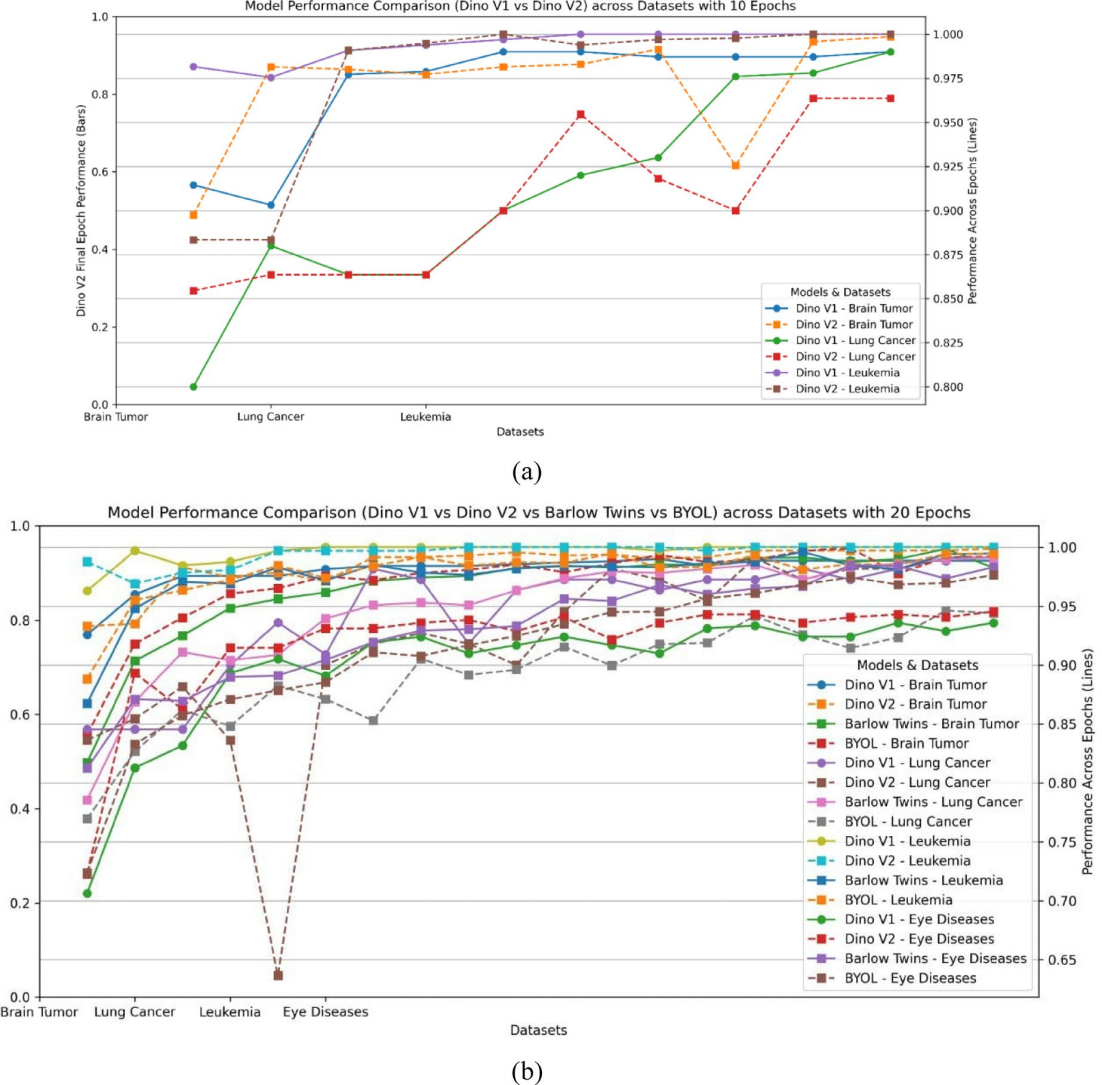
Comparison between SSL models (**a**) with 10 epochs and (**b**) with 20 epochs.

**Fig. 12:**
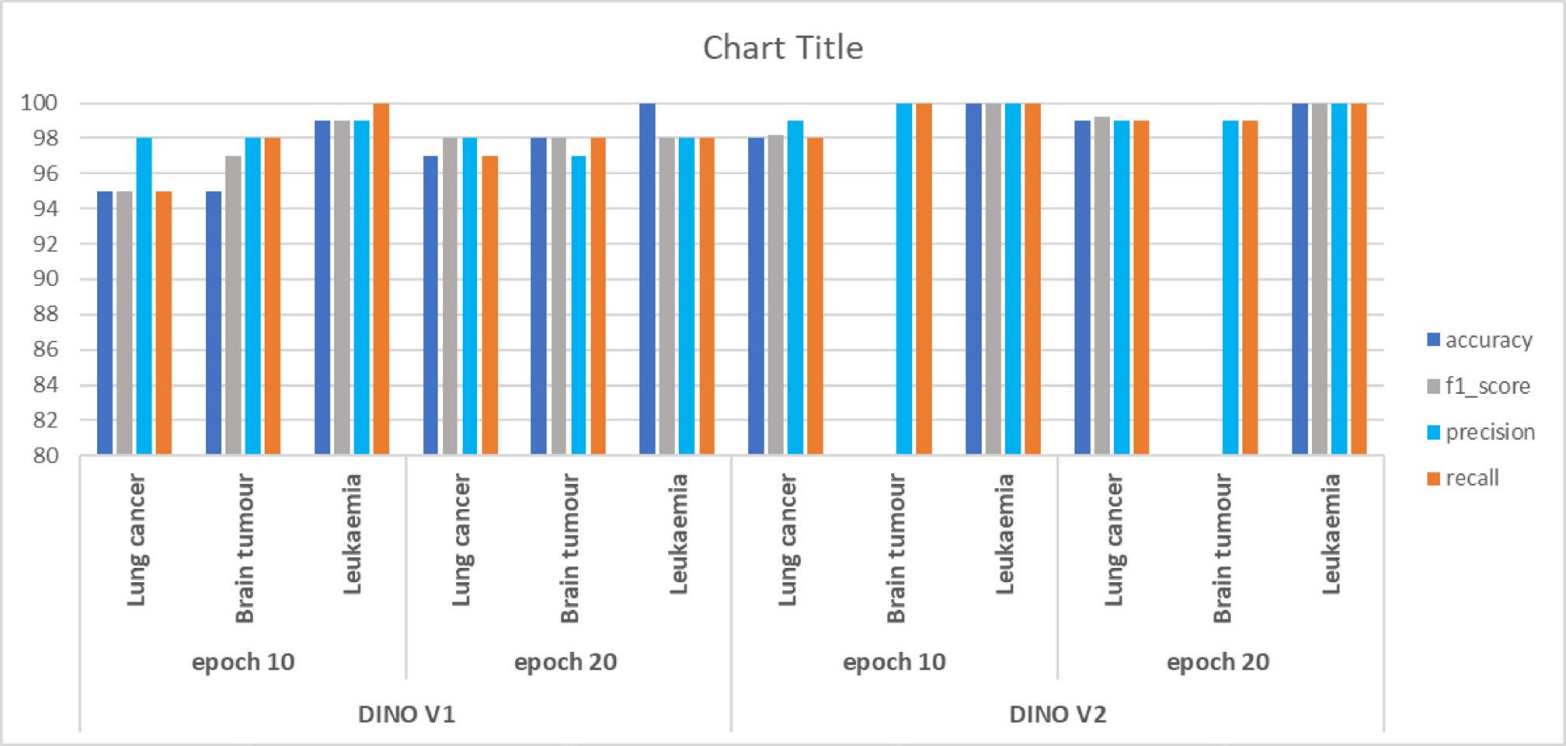
Comparison between SSL models in terms of all evaluation metrics.

When comparing the two learning approaches, it is evident that utilizing self-supervised learning, as exemplified in DINO, surpasses traditional supervised learning in medical image classification. This is attributed to the structure of the DINO model, which permits the extraction of meaningful features from the training data in a way that contributes to enhancing overall performance. These findings promise to improve medical diagnosis efficiency through a promising research direction in the healthcare domain. Figure 11 shows a comparison between SSL models (a) with 10 epochs and (b) with 20 epochs, and Fig. 12 shows a comparison according to all evaluation metrics.

### Statistical test

Statistical tests are critical in comparing various models across the same datasets^44^. The Friedman test and the Nemenyi test (post-hoc) are utilized to identify the specific test between groups. The Friedman test first ranks the data within the blocks, while the Nemenyi test compares all groups to identify the pairs of blocks that specify the significant variations^45^. The critical difference^46–48^ The threshold used in post hoc tests specifies whether the difference is statistically significant. If the difference between s is more than the critical difference (CD), it is considered important. This method is utilized. As shown in the figures, for lung cancer, brain tumor, and Leukemia, DINO V1 with 20 epochs gives the best results. For the retina dataset, DINO V2 with 20 epochs gives the best performance. This result confirms our hypothesis that states that self-supervised learning outperforms traditional supervised learning.

### Semantic search

Semantic search^1^ is a valuable technique in medical imaging that enables the identification of patients with similar illnesses and the extraction of similar images, significantly facilitating prompt and precise diagnosis. We use Qdrant to perform semantic searches across our database and extract image embeddings using the DINO model, as referenced in Table 1. This method improves patient outcomes by optimizing the retrieval and analysis of medical images, enabling medical professionals to obtain relevant case studies and image data quickly and effectively.

#### Image embeddings

Using the Vision Transformer (ViT)^2^, we extract meaningful features from pictures and store them in a vector database. Image embeddings are numerical representations of images that capture their semantic content, allowing the system to comprehend and compare images based on their attributes rather than pixel-level information. These derived features enhance the efficiency and accuracy of multiple computer vision tasks, including object detection, image classification, and image retrieval.

#### Qudra net dataset

Quadrant is a vector database and search engine designed to handle high-dimensional data such as vectors or embeddings for open-source use. It aims to make similarity and nearest neighbour searches more efficient, which are crucial for tasks like semantic search.

Image embeddings are extracted from the Vision Transformer model and stored in Quadrant. Then, a semantic search is performed to find similar images or related cases within the database.

**Fig. 13:**
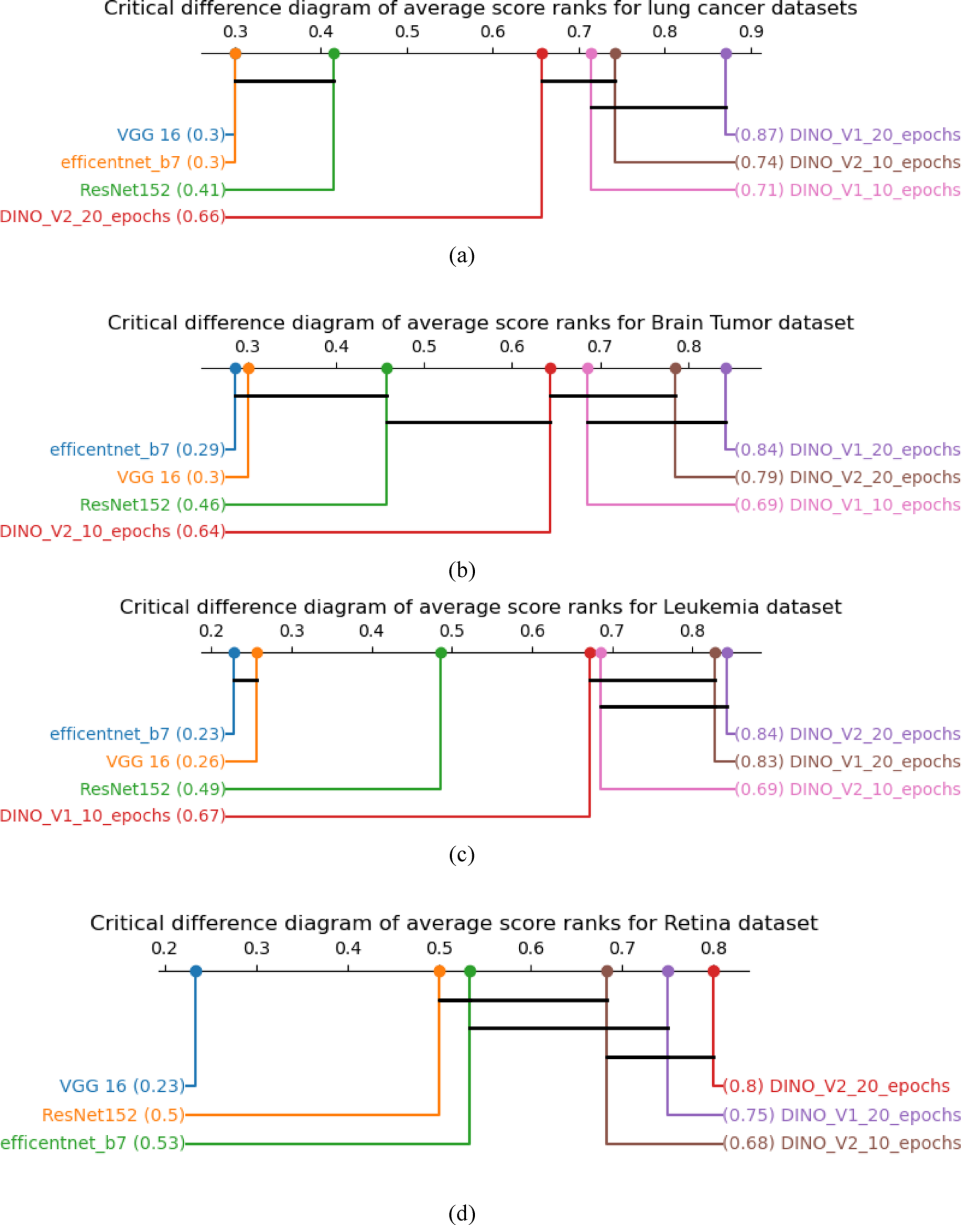
critical difference (**a**) for lung cancer dataset, (**b**) for brain tumor dataset, (**c**) for leukemia dataset, and (**d**) for retina dataset.

The image is first converted into an embedding vector when a query is made. This vector is then compared to the vectors in the database to find similar images, as shown in Figs. 12 and 13. The typical method for comparing two vectors is to use cosine similarity. This metric measures the cosine of the angle between them, which is defined as:

5 $$\text{Cosine Similarity}=\frac{A.B}{|\left|A\right|| ||B||}$$

**Fig. 14:**
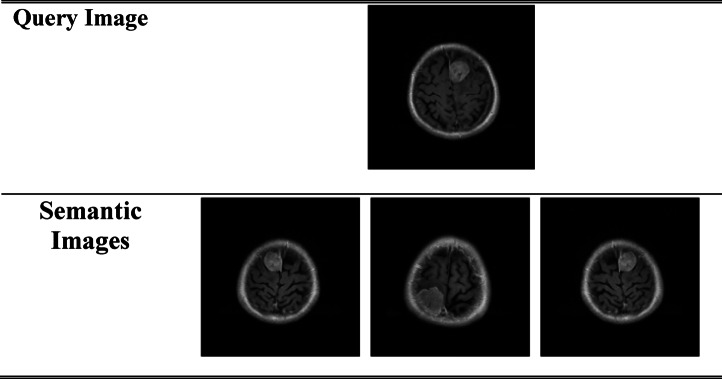
Semantic search for brain tumour.

**Fig. 15:**
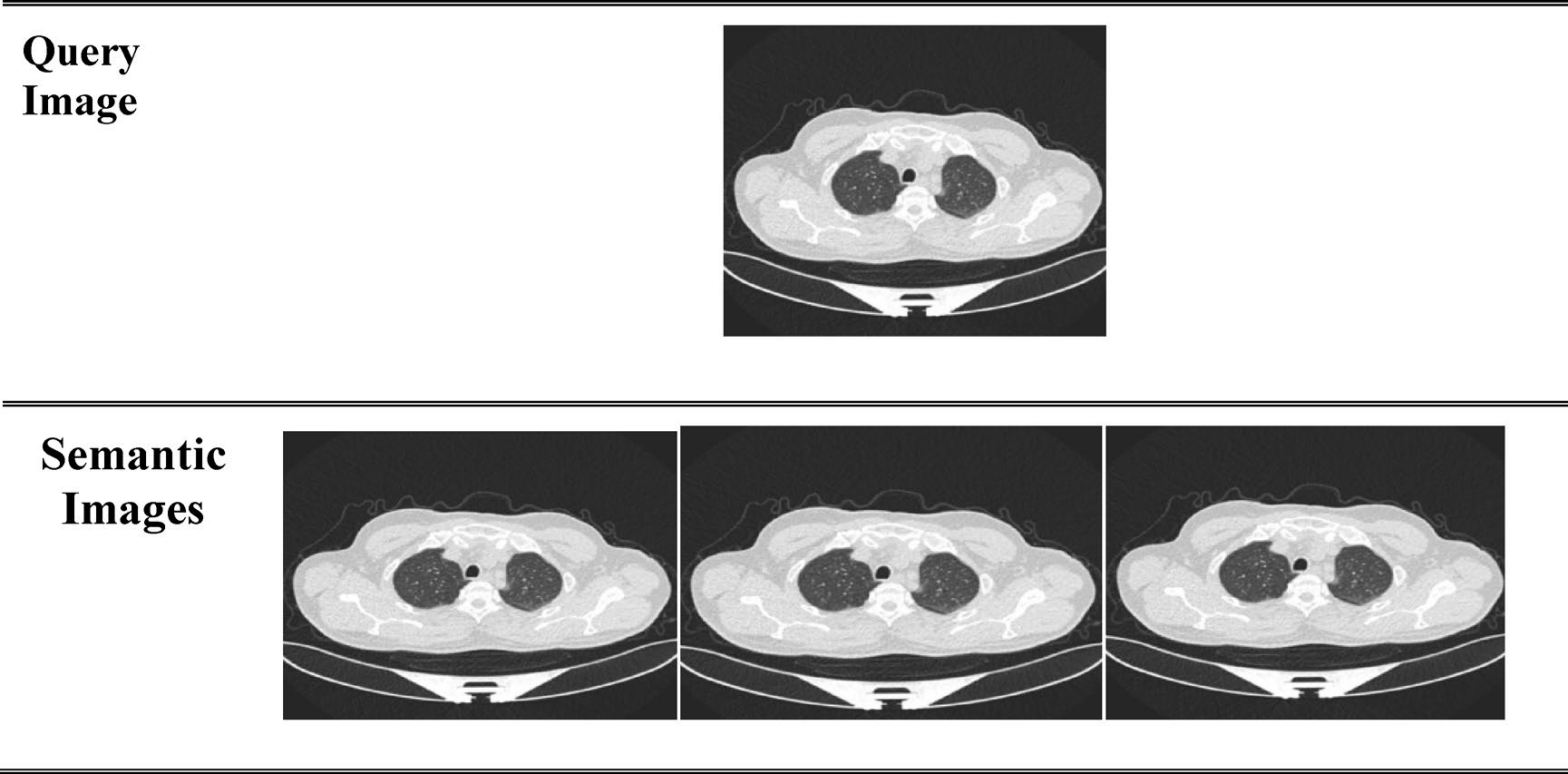
Semantic search for lung cancer.

A and B are the embedding vectors of the query and database images, respectively. A cosine similarity close to 1 indicates that the images are highly similar, while a value closer to 0 means less similarity. Figure 14 shows the query images of the brain tumour, and Fig. 15 shows the Semantic Search results for Lung Cancer. To measure the relevance of the retrieved images. We utilized percsion@k, which is the information retrieval metric that measures the proportion of the relevance of the top-k retrieved results. It is calculated through the following equation

6 $$Percsion@k= \frac{Number\;of\;relevant\;itmes\;in\;the\;top\;k\;reult}{k}$$

Our DINO v2 proposed model achived promising performance across the quanative metric (1) the top_1 accuracy 89.2% (exact match) and top_5 accuracy of 96.7% on test queries (2) periscion@k values of 0.89 (P@1), 0.88 (P@2), and 0.83 (P@5) which outperforms traditonal search by 26–39%.

### Model explanation

In medical image classification, the explanation is critical for enhancing model transparency and providing insights that help the model. This step concentrates on making the model’s predictions more interpretable and transparent. Our paper generates visualisations highlighting the most influential parts of the model’s decision-making process.

The following figures demonstrate the ViT CX explanation applied to the DINO V2 vision transformer model for three cancer classification tasks. Each figure shows a sample from a specific class, with the original medical image on the right and the corresponding heatmap on the left. These heatmaps visualise DINO V2’s attention distribution, with red areas indicating the highest importance and the blue regions least significant for the model’s decision-making. This visualisation provides insights into DINO V2’s classification strategy, highlighting key distinguishing features between cancer types and stages. Given the sensitive nature of cancer diagnosis, understanding the model’s decision-making process is crucial for building trust in AI-assisted medical image analysis. By analyzing these heatmaps alongside the original images, we can better understand the model’s focus, identify clinically relevant features, and ensure the model’s reliability in this critical healthcare application.

#### How Vit-CX is applied to DINOv2

Vit-CX is a causal explainability framework designed explicitly for Vision Transformers, enabling a more principled analysis of model decision-making by identifying causal relationships between input features and predictions. Unlike traditional attention-based visualization techniques, Vit-CX disentangles correlation from causation by leveraging counterfactual interventions.

Our study integrates Vit-CX with DINOv2 to generate explanations for cancer classification. The method perturbed different image regions and assessed their impact on the model’s decision. The causal contribution of each region is then visualized using heatmaps, where red regions indicate strong causal influence, while blue regions indicate minimal impact.

To apply Vit-CX, we first preprocess medical images by normalizing intensity values and resizing them to match the input dimensions of DINOv2. The model’s attention weights are extracted and analyzed using the ViT-CX framework, which iteratively perturbs key regions and observes changes in classification confidence. This approach allows us to generate fine-grained explanations of what the model considers crucial for its predictions.

#### Example application of ViT-CX on lung cancer dataset

To illustrate the process of ViT-CX, we analyze a lung CT scan classified as malignant. Initially, the image is divided into patches, and each patch’s embedding is extracted. ViT-CX then applies a masking strategy, systematically altering different patches to observe how these changes influence the model’s prediction.

In our experiment, the saliency map produced by ViT-CX highlights a large red region in the right lung, corresponding to the tumour location. Unlike traditional Grad-CAM, which assigns importance based on attention scores, ViT-CX refines the explanation by correcting spurious correlations, ensuring that only patches with an actual causal impact on the decision remain highlighted. This improves trust in the AI system by making the model’s decision process more transparent and more reliable.

#### Explanation of lung cancer dataset

**Fig. 16:**
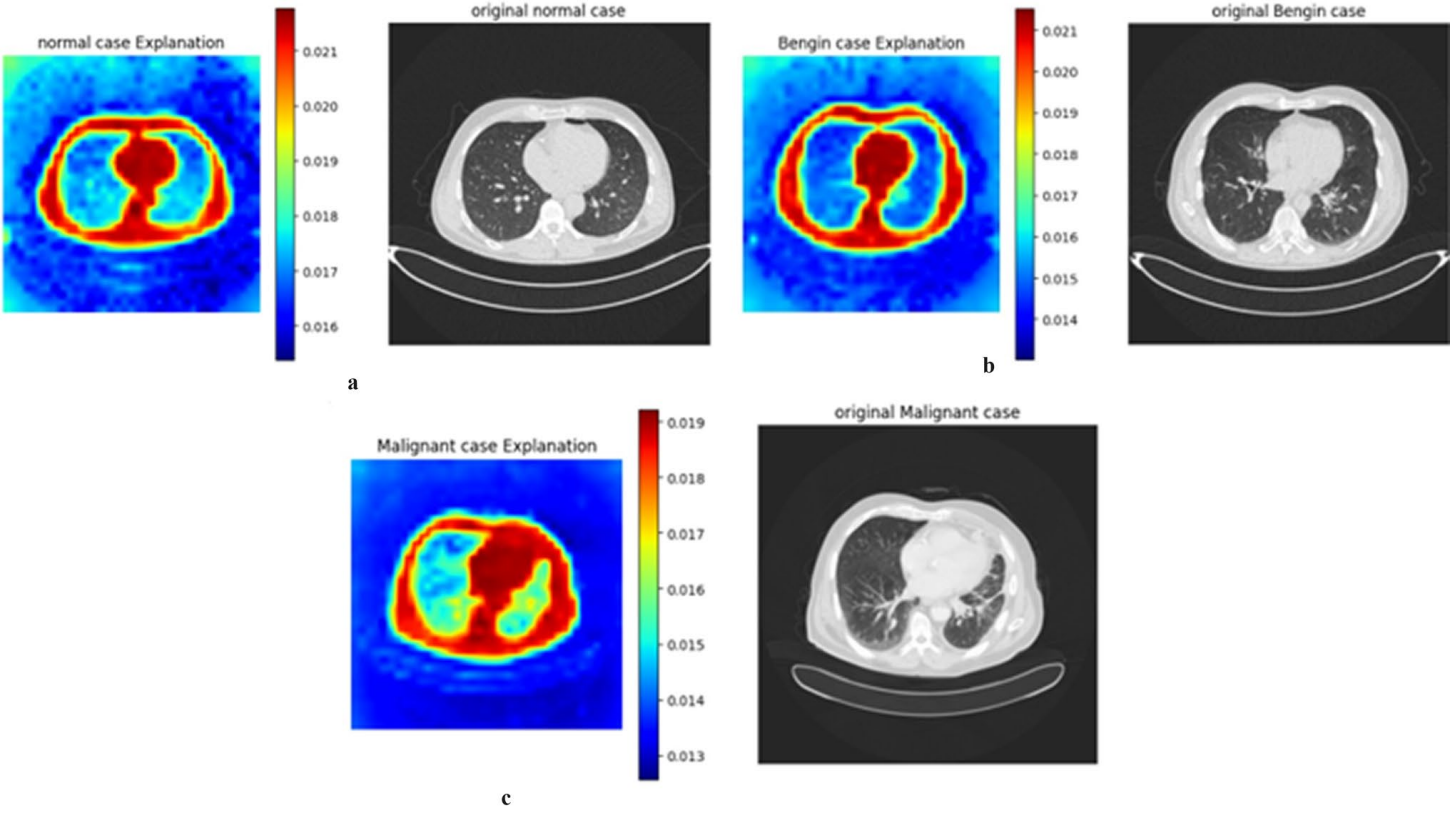
Explanation of lung cancer classification. (**a**) Normal class; (**b**) Benign class; (**c**) Malignant.

As shown in Fig. 16a, the heatmap for the normal cases shows high importance with red around the lung borders and central structures. This suggests that the model focuses on the overall lung shape and the absence of abnormalities in these areas to classify a scan as normal.

As shown in Fig. 16b, the heatmap for the benign case highlights the lung borders in red color and some internal structures in green and yellow, which is more intense than in the normal case. This could indicate that the model detects subtle changes associated with benign conditions, particularly in the lung’s periphery.

As shown in Fig. 16c, the heatmap for the malignant case shows a large area of high importance with red in the right lung corresponding to the tumor’s location in the CT scan. This demonstrates the model’s ability to identify and focus on areas with potential malignancy.

#### Explanation of leukemia dataset

**Fig. 17:**
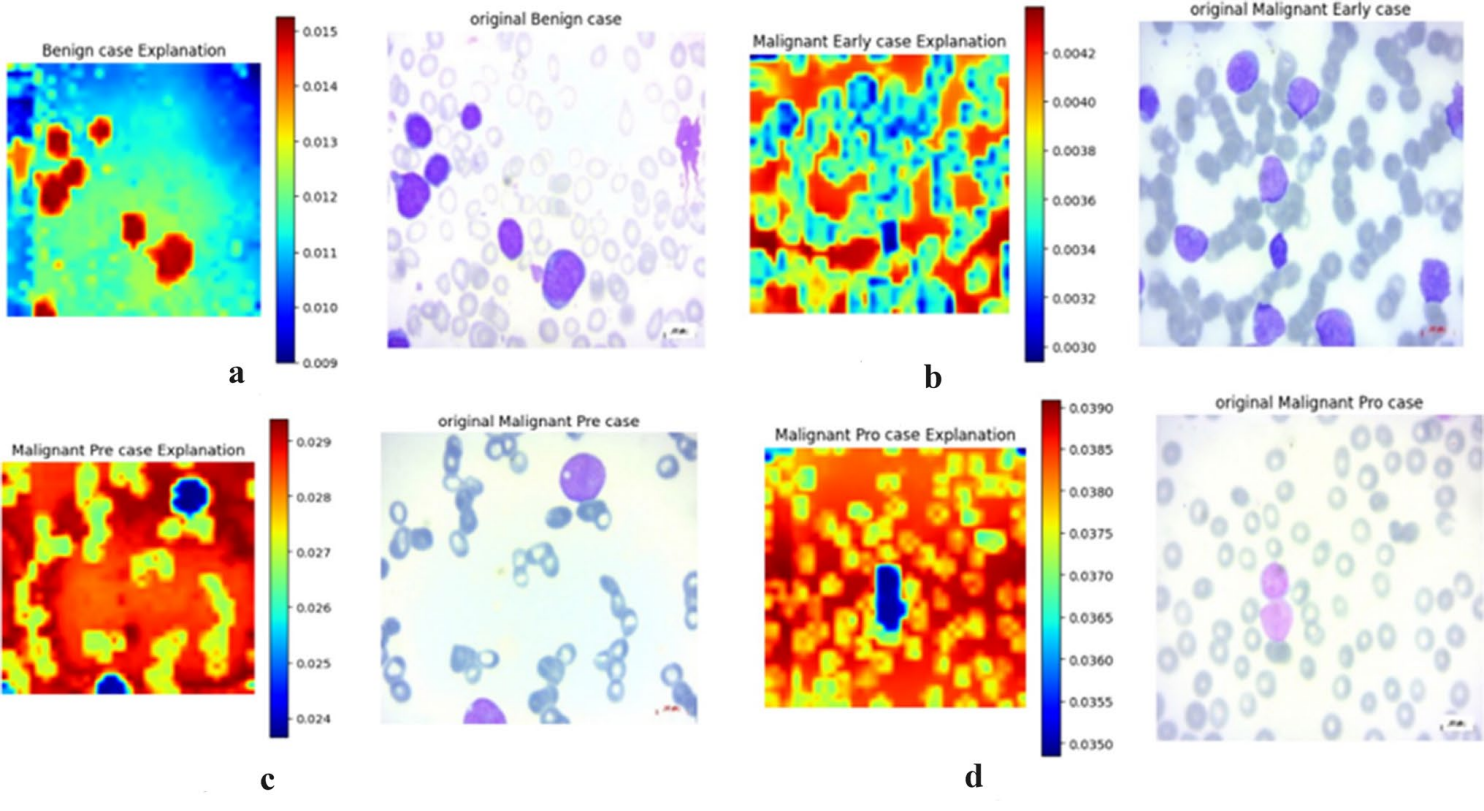
Explanation of leukemia classification. (**a**) Benign class; (**b**) Malignant early; (**c**) Malignant pre; (**d**) Malignant pro.

In Fig. 17a, the benign case heatmap shows concentrated areas of high importance in red corresponding to the larger, purple-stained cells in the original image. This suggests that the model focuses on individual cells’ size and staining intensity when classifying a sample as benign. The model appears to pay less attention to the smaller, lighter-stained cells, which are indicated by green and blue.

The early malignant case heatmap (Fig. 17b) reveals an intriguing pattern. The model highlights the spaces between the small cells in red while giving less attention, indicated by green, to the cells themselves. This suggests that the model detects subtle changes in cellular arrangement and spacing, which may be crucial for identifying early signs of malignancy. The small cells in the original image appear to form specific shapes or patterns, and the model seems particularly attuned to these intercellular spaces.

For the pre-malignant case (Fig. 17c), a pattern like the early malignant case, but with some key differences. The cells again form patterns, but the spaces between them appear larger, highlighted in red, and the model shows high importance to them. The pattern of the small cells, highlighted in green, is less important, while the large purple cells, highlighted in blue, are unimportant to the model.

The pro-malignant case heatmap (Fig. 17d) shows a unique pattern where most of the field is highlighted in red but with a distinct blue area corresponding to a larger, darker-stained cell in the original image. This suggests that the model is looking at individual cell characteristics and the relationships between cells and their surrounding environment. The high importance placed on the areas surrounding the darkly stained cell, rather than the cell itself, suggests that the model is detecting disruptions in normal cellular arrangements, which are characteristic of advanced malignancy.

#### Explanation of brain tumour dataset

**Fig. 18:**
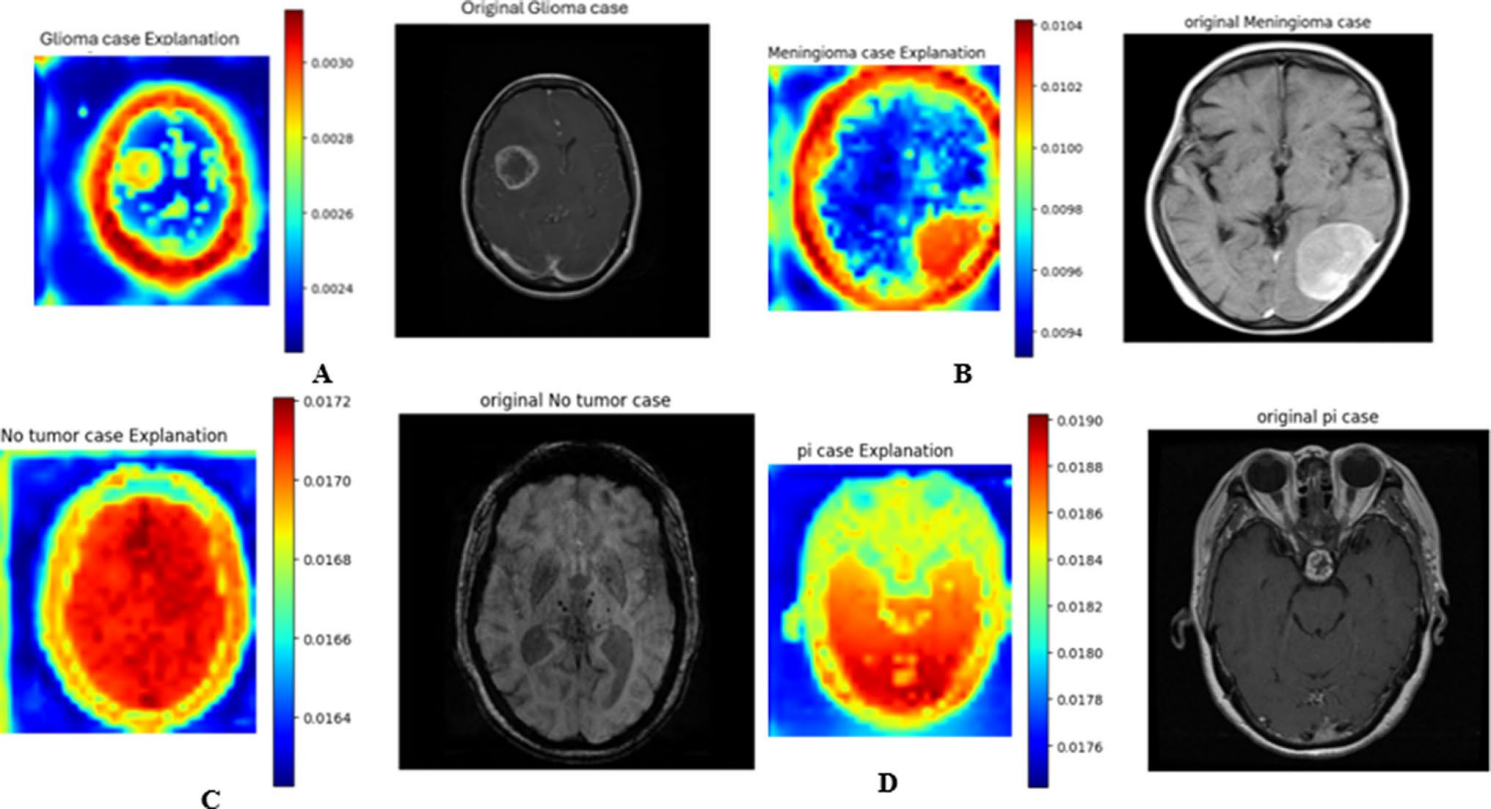
Explanation of brain cancer classification. (**A**) glioma class, (**B**) meningioma class, (**C**). no tumor class, (**D**) pituitary class.

In Fig. 18A, the glioma case heatmap shows concentrated areas of high importance highlighted in red and yellow corresponding to the tumour location in the original MRI image. The bright red areas indicate the model focuses intensely on the tumour’s borders and internal structure. Green and blue represent the surrounding brain tissue, indicating less importance in the classification decision.

The meningioma case heatmap (Fig. 18B) reveals that the tumour area is highlighted in red, and significant attention is paid to the brain’s outer contours and the area adjacent to the tumour in red and yellow. This suggests that the model is not only focusing on the tumor itself but also on its relationship to surrounding structures. It is crucial to identify meningiomas that often arise from the meninges and can cause displacement of adjacent brain tissue.

In the no tumor case (Fig. 18C), the heatmap shows the central regions of the brain are highlighted in red, while the peripheral areas are in yellow. This suggests that the model examines the brain’s overall structure and symmetry to confirm the absence of tumours. The focus on central structures may indicate that the model particularly attends to areas where tumors commonly occur.

The pituitary case heatmap (Fig. 18D) shows a unique pattern where the highest importance in red colour is placed on the lower central region of the brain, corresponding to the location of the pituitary gland. The model’s attention, as indicated by the red and yellow areas, extends beyond the immediate pituitary region, possibly capturing the effects of the tumor on surrounding structures. This highlights the model’s ability to focus on the specific **anatomical** location characteristic of pituitary tumors.

#### Interpretation of results

The heatmaps produced by ViT-CX provide a deeper understanding of DINOv2’s decision-making by highlighting regions that directly influence the classification outcome. Compared to traditional methods like Grad-CAM, which primarily highlight attended areas, ViT-CX ensures that the identified regions are causally linked to the final prediction rather than merely being correlated.

For instance, in lung cancer classification, ViT-CX not only highlights tumor regions but also captures the absence of abnormalities in normal cases, reinforcing its ability to explain both positive and negative decisions. Similarly, in leukemia classification, the model appears to focus on the arrangement of cells rather than isolated features, suggesting that it learns structural relationships crucial for distinguishing malignancy stages.

## Conclusion

Medical images are considered an important source of information. However, their reliance on labeled data limits their applicability in various clinical settings. As a result, our study mainly concentrates on tackling these challenges by exploring the performance of SSL in diverse datasets and comparing it with the performance of traditional SL models. Our study utilizes both DINO V1 and DINO V2 to surmount the labeling issue of the SL. The results assured the ability of SSL to overcome the challenges of SL with superior performance in diverse medical datasets. To ensure model transparency, we utilize explainable AI, highlighting the images’ areas that lead to the final decision. Explainable AI improves trust in the model decision and facilitates the integration of the developed model in the clinical workflow.

Furthermore, the research investigates the ability to retrieve images with the same decision and semantic searchability. First, we extracted the model embeddings from the DINO model and then saved it in the Qudra Net database. Semantic search using cosine similarity was utilized to retrieve images similar to the query image. Our research aims to provide a comprehensive system that can take advantage of unlabeled medical images in terms of classification, explanation, and retrieval. We plan to build a comprehensive system that uses the proposed healthcare models. Unless the superiority of our proposed model. Some limitations need to be handled, including: (1) We focused on four cancer types (lung, brain, leukaemia, retina) from the single-centre dataset. This helps control comparisons (As in Tables 2 and 3); the developed results may not generalize to other cancer types (e.g., breast, prostate) (2). Utilizing the geometric transforms only for data augmentations could limit robustness to scanner variation with different intensities. Accordingly, we intend to try different augmentation techniques. To validate ViT-CX’s clinical utility, Table 4 includes a comparison between ViT-CX, Grad-CAM, and Attention using specific criteria, including the following (feature localization, clinical coherence. This comparison showed that ViT-CX’s causal perturbation approach eliminated Grad-CAM’s.

**Table 4:** Comparison between explanation techniques.

	Feature localization	Clinical coherence		Computational cost
		Radiologist agreement	Pathology alignment	
ViT-CX’s	Highlighted diagnostically relevant regions	92%	89%	ViT-CX added 15ms
Grad-CAM	Often included adjacent healthy tissue (↑ false-positive regions by 23%)	74%	68%	Less than Vit_CX interms of time
Attention	Showed diffuse attention patterns	81%	72%	

## Data Availability

Data is available on request due to ethical restrictions. Contact with corresponding author to request the data.
